# Trichocystatin-2 from *Trichomonas vaginalis*: role of N-terminal cysteines in aggregation, protease inhibition, and trichomonal cysteine protease-dependent cytotoxicity on HeLa cells

**DOI:** 10.3389/fpara.2025.1512012

**Published:** 2025-03-18

**Authors:** Verónica Aranda-Chan, Montserrat Gutiérrez-Soto, Claudia Ivonne Flores-Pucheta, Octavio Montes-Flores, Rossana Arroyo, Jaime Ortega-López

**Affiliations:** ^1^ Departmento de Biotecnología y Bioingeniería, Centro de Investigación y de Estudios Avanzados del Instituto Politécnico Nacional (Cinvestav), Mexico City, Mexico; ^2^ Departamento de Infectómica y Patogénesis Molecular, Centro de Investigación y de Estudios Avanzados del Instituto Politécnico Nacional (Cinvestav), Mexico City, Mexico

**Keywords:** trichocystatin-2, *Trichomonas vaginalis*, protein aggregation, cysteine protease inhibitors, cytotoxicity

## Abstract

*Trichomonas vaginalis* is a protozoan parasite that causes trichomoniasis, the most common nonviral neglected sexually transmitted disease worldwide. Biomarkers and therapeutic targets, including specific trichomonad cysteine proteases (CPs) and their endogenous inhibitors, have been identified to diagnose and treat this disease. Trichocystatin 2 (TC-2) was previously identified as one of the three endogenous inhibitors of the parasite’s cathepsin L-like CPs, including TvCP39, which is involved in *T. vaginalis* cytotoxicity and is a potential therapeutic target. TC-2 contains five cysteines, including four located in the N-terminal sequence. These cysteines may be responsible for the formation of multimers of the recombinant protein expressed in *E. coli*. To determine whether these cysteines are responsible for the formation of TC-2 multimers and the effect of the N-terminus on CP inhibition, a recombinant TC-2 mutant was expressed, purified, characterized, and compared with the recombinant wild-type TC-2 protein. *In silico* and experimental analyses revealed that wild-type and mutant TC-2 proteins presented similar results in terms of secondary and tertiary structure prediction and high thermal stability. However, compared with that of wild-type TC-2, multimer formation was significantly reduced in the mutant lacking the four N-terminal cysteines, leading to a significant reduction in papain inhibition but not in trichomonal CP activity. These results support the hypothesis that the four cysteines located in the N-terminal region are responsible for aggregation, and their deletion affected the interaction of TC-2 with papain without affecting its inhibitory activity on homologous target proteases that are crucial for *T. vaginalis* virulence. Our results provide essential data supporting the use of TC-2 as a potential therapeutic target.

## 1 Introduction

*Trichomonas vaginalis* (*T. vaginalis*) is a parasitic protozoan responsible for trichomoniasis, a common neglected sexually transmitted infection, with a prevalence of at least 156 million cases per year (WHO, 2023). *T. vaginalis* infection is associated with adverse pregnancy outcomes, including increased risks of preterm birth, premature rupture of membranes, and small for gestational age infants (Silver et al., 2014; Margarita et al., 2020). Additionally, this pathogen has significant public health implications, particularly in terms of its interactions with other infections, such as human immunodeficiency virus (HIV) (Kissinger, 2015). Thus, the identification of possible pharmacological or diagnostic targets is essential to treat this disease more efficiently. *T. vaginalis* cysteine proteases are a major type of parasite protease and have been identified as potential therapeutic targets given their association with parasite virulence (Arroyo et al., 2015). During the study of cysteine proteases (CPs), the endogenous inhibitor trichocystatin-2 (TC-2), a 10-kDa cystatin protein of the stefin type located in the cytoplasm and lysosomes associated with TvCP39, was identified. TC-2 inhibits the activity of papain and cathepsin L as well as the proteolytic activity of *T. vaginalis* protease-resistant extracts (PRE), and was even able to protect HeLa cell monolayers from cytotoxic damage caused by the parasite (Puente-Rivera et al., 2014).

Cystatins are a superfamily of CP reversible inhibitors found in many organisms, including protozoans, plants, animals, and humans. They play crucial roles in regulating protease activity, which is essential for maintaining different physiological processes (Wickramasinghe et al., 2020). Cystatins are classified into three main types: stefins (type I), cystatins (type II), and kininogens (type III). Stefins are mainly intracellular, whereas cystatins and kininogens are extracellular (Turk et al., 2008; Magister and Kos, 2013). They share conserved structural motifs, including the cystatin-like domain, and specific sequences such as QXVXG (Wickramasinghe et al., 2020). In addition, cystatins also have potential as biomarkers for various diseases and as targets for developing new therapeutic drugs (Turk et al., 2008; Breznik et al., 2019). Moreover, parasite-derived cystatins have potential therapeutic applications in treating immune-mediated disorders because of their immunomodulatory properties. Additionally, they can induce anti-inflammatory responses and be developed as biotherapeutic agents (Khatri et al., 2020).

Therefore, TC-2 is regarded as a protein warranting further characterization given its essential role in trichomoniasis. For this purpose, its recombinant production is needed. However, when TC-2 is recombinantly expressed in *E. coli*, it is produced as multimeric aggregates, which could impair its stability and make it challenging to work with when a larger scale and longer production time are needed. Thus, given that TC-2 possesses five cysteines, including four located at the N-terminus, these cysteines are proposed to promote the formation of recombinant TC-2 multimers. In this study, we demonstrate the involvement of the N-terminal cysteines of TC-2 in aggregation and decipher the role of the N-terminus in inhibiting the proteolytic activity of CPs from *T. vaginalis*.

## 2 Materials and methods

### 2.1 *In silico* docking

The amino acid sequences of TC-2 (TVAG_272260) and the TC-2Δ11 deletion mutant, as well as the target CPs TvCP2 (TVAG_057000) and TvCP39 (TVAG_298080) (https://trichdb.org/trichdb/app/) (Release 63 01 May 2024) (Alvarez-Jarreta et al., 2024) and papain (PDB:1CVZ) (Tsuge et al., 1999), were modeled using the AlphaFold3 structure prediction server (https://alphafoldserver.com/) (Abramson et al., 2024). The subsequent analysis, preparation, and visualization of the models were performed using ChimeraX software (https://www.rbvi.ucsf.edu/chimerax) (Meng et al., 2023). The models were validated using the MolProbity server (http://molprobity.biochem.duke.edu/index.php) (Williams et al., 2018). Molecular docking of each of the inhibitors (TC-2 and TC-2Δ11) with each target CP was performed using the AlphaFold3 server (Abramson et al., 2024), Prodigy server (https://rascar.science.uu.nl/prodigy/) (Xue et al., 2016) and Area Affinity server (https://affinity.cuhk.edu.cn/) (Yang et al., 2022).

### 2.2 Recombinant expression of rTC-2 and rTC-2Δ11 in *E. coli*

The TC-2 gene was previously cloned within the expression vector pCold I. This construct was subsequently transformed into *E. coli* BL21 (DE3), and its expression was induced as previously reported (Puente-Rivera et al., 2014). For the expression of rTC-2Δ11, the TC-2 gene sequence (TVAG_272260) was modified by deleting its first 11 amino acids, which include the four N-terminal cysteines. The DNA sequence was optimized for its expression in *E. coli* and synthesized by Synbio Technologies (LLC, NJ, USA). It was cloned between the *Nco*I and *Xho*I restriction sites within pUC57. The optimized sequence was subsequently subcloned and inserted into the pCri8a expression vector (Addgene plasmid # 61317; http://n2t.net/addgene:61317; RRID: Addgene_61317) (Goulas et al., 2014). The pCri8a construct was transformed into *E. coli* BL21 (DE3), and clones were analyzed by plasmid double digestion with the *Nco*I and *Xho*I restriction enzymes and selected for subsequent protein expression. The expression of both proteins was first induced in a flask culture. Transformed *E. coli* BL21 (DE3) clones were grown in 0.5 L of Luria–Bertani (LB) media (casein peptone 10 g/L, yeast extract 5 g/L, NaCl 10 g/L) or yeast extract tryptone media (2TY) (16 g/L tryptone, 10 g/L yeast extract, 5 g/L NaCl) supplemented with ampicillin (100 µg/mL) or kanamycin (50 µg/mL) as selective antibiotics. The bacterial culture was grown at 37°C and 200 rpm until the optical density (OD_600_) reached 0.6. Recombinant protein expression was induced by adding a final concentration of 0.2 mM β-D-1-thiogalactopyranoside (IPTG) for 12 h with the culture maintained at 30°C and 200 rpm.

### 2.3 Purification of the recombinant proteins rTC-2 and rTC-2Δ11.

To purify the recombinant proteins from a single production batch, the expression of rTC-2 and rTC-2Δ11 was scaled up to a 3 L jar bioreactor (BioFlo/Celligen 310 controller, Eppendorf) using a fed-batch culture. Briefly, the volume of medium necessary to inoculate a bioreactor was taken from a seed inoculum. Fermentation was started with an OD_600_ of 0.05 in 2 L of BSM medium (3.5 g/L KH_2_PO_4_, 5 g/L K_2_HPO_4_, 3.5 g/L (NH_4_)_2_HPO_4_, 4 mL/L 1M MgSO_4_, 20 g/L glycerol, 5 g/L yeast extract, and 1 mL/L trace metals) with the appropriate antibiotic added for the selection of each protein and 0.5 mL/L of 10% Antifoam 204 (Sigma). The culture was grown at 37°C for approximately 12 h until glycerol was depleted. Protein expression was induced with 0.5 mM IPTG for 16 h at 18°C, and 50% glycerol was fed simultaneously at a rate of 3 mL/L*h.

Biomass was recovered by centrifugation in Sorvall LYNX (Thermo Fisher Scientific, USA) at 4248× g for 30 min. The induction of expression was validated using 15% SDS−PAGE. Lysis was then performed by adding 20 mL of lysis buffer (50 mM Tris-HCl [pH 8.0], 500 mM NaCl, 5 mM imidazole, 10% glycerol, and 0.02% sodium azide) per gram of wet biomass, followed by the addition of lysozyme to a final concentration of 0.5 mg/mL and incubated for 30 min at 37°C and 220 rpm. Then, the sample was immersed in an ice water bath (4°C) and sonicated using a 550 Sonic Dismembrator (Thermo Scientific, USA) at 30% of the wave amplitude by 6 x 30 s pulse with 30 s interval between each sonication cycle. The soluble fraction was recovered by centrifugation at 20 216 × g for 30 min at 4°C. Purification was performed by affinity chromatography on prepacked Ni-Sepharose columns (Cytiva, USA). The column was equilibrated with lysis buffer, and the protein was eluted with elution buffer (50 mM Tris-HCl [pH 8.0], 500 mM NaCl, 500 mM imidazole, 10% glycerol, and 0.02% sodium azide). For long-term storage at -80°C, sterile glycerol was added to the protein mixture at a final concentration of 25%. The final protein concentration was determined by a bicinchoninic acid (BCA) assay (Thermo Fisher, USA). The samples were analyzed by 15% SDS−PAGE under reducing or nonreducing conditions. Recombinant proteins were subjected to dynamic light scattering (DLS), thermal shift (TS), and size exclusion chromatography (SEC) after a buffer exchange (50 mM Tris-HCl pH 8.0, 100 mM NaCl, 0.02% NaN_3_) with gel filtration on PD-10 columns (Cytiva, UK).

### 2.4 Western blot analysis

Immunodetection of rTC-2 and rTC-2Δ11 was performed by Western blot (WB) assays, as previously reported (Puente-Rivera et al., 2014). Briefly, 4 µg rTC-2 and rTC-2Δ11 proteins were separated by 15% SDS-PAGE, and transferred onto a nitrocellulose (NC) membrane. The NC membrane was blocked with 5% skim milk for 18 h at 4°C and incubated with the Rα-rTC-2 primary antibody (1:500), as previously described (Puente-Rivera et al., 2014); washed with Tris-buffered saline (TBS, 20 mM Tris-HCl, 500 mM NaCl)-0.5% Tween 20; and incubated with a goat anti-rabbit peroxidase-conjugated secondary antibody (1:3000) (Bio-Rad, USA). Protein detection was performed by chromogenic method with 4-chloro-naphtol (0.5 mg/mL) in TBS and 0.05% H_2_O_2_.

### 2.5 Hydrodynamic diameter measurement using dynamic light scattering

The hydrodynamic diameter (Dh) of both recombinant proteins was determined using DLS under reducing (1, 5, or 15 mM DTT) or nonreducing conditions for samples after 1, 3, and 6 months of storage at 4°C. Samples of recombinant proteins (1 mg/mL) on storage buffer (50 mM Tris-HCl pH 8.0, 100 mM NaCl, 0.02% NaN_3_) were centrifuged at 17 949 *× g* for 10 min (4°C) and filtered through 0.22 µm membrane. Measurements were performed using a Zetasizer nano ZSP (Malvern Panalytical, USA) in 173° backscatter mode at 25°C with a 40 µL cuvette [ZEN0040] (Brand, Germany). Two replicates were obtained for each sample, and at least three data acquisitions were conducted. The data were analyzed using the Zetasizer software (v.7.12; Malvern Instruments).

### 2.6 Stability analysis using TS assay

The TS assay was performed in 50 µL of Tris buffer (50 mM Tris-HCl pH 8.0, 100 mM NaCl, 0.02% NaN_3_) containing 20 µM protein and 1x Sypro orange (Invitrogen, USA). Assays were performed using a real-time qPCR Gentier 48E thermal cycler (Tianlong, China). The temperature gradient was adjusted from 25 to 95°C with 1°C/min increments. Each sample was run in triplicate.

### 2.7 Molecular weight estimation using size exclusion chromatography

The molecular weight and size were determined using analytical SEC for rTC-2 and rTC-2Δ11 in samples stored for 1 and 3 months. SEC was performed using a Superdex 200 pg HiLoad 26/600 column (GE Healthcare Bioscience, Sweden) on an ÄKTA Pure 25 system (GE Healthcare, USA). Before sample injection, the column was calibrated with gel filtration standards [1511901] (Bio-Rad). Five milligrams of protein were loaded on three runs, and the proteins were resolved with elution buffer (50 mM Tris-HCl, pH 8.0; 100 mM NaCl; and 0.02% NaN_3_ at a velocity of 29.38 cm/h). The results were analyzed with Unicorn software (v7.1, GE Healthcare, USA).

### 2.8 Inhibition of the proteolytic activity of papain and *T. vaginalis* protease-resistant extracts (PRE) by rTC-2 and rTC-2Δ11

The ability of rTC-2 and rTC-2Δ11 to inhibit protease activity was evaluated using papain and *T. vaginalis* PRE, and the fluorogenic substrates Z-Phe-Arg-MCA (Sigma) and E-64 (trans-Epoxysuccinyl-L-leucylamido(4-guanidino)butane) as CP control inhibitor. Papain (2 ng/µL) was activated with buffer (50 mM Tris, pH 6.5; 5 mM DTT) for 10 min at 25°C and incubated with rTC-2, rTC-2Δ11, or E-64 (0.7 µm). The reaction started upon the addition of the fluorogenic substrate Z-Phe-Arg-MCA (40 µM), as previously reported (Puente-Rivera et al., 2014). The fluorescence was measured at an excitation wavelength (λ) of 355 nm and emission wavelength (λ) of 460 nm on a SpectraMax Gemini EM spectrofluorometer (Molecular Devices). For the inhibition assays of PRE proteolytic activity, 2 x 10^7^ parasites from the CNCD 188 trichomonad isolate resuspended in a PBS pH 8.0 were lysed with 0.5% sodium deoxycholate (DOC) in the absence of protease inhibitors and centrifuged in a 10% sucrose gradient at 16 200 *x g*, for 30 min at 4°C. The supernatant was recovered, and PRE protein concentration was estimated at A_280_ against a previous calibration curve. Immediately, the inhibition assays were done as described for papain by using 20 µg of PRE incubated in activation buffer (50 mM NaOAc, pH 5.0; 4 mM EDTA; and 8 mM DTT) with different concentrations (0 µM, 0.7 µM, 2 µM, or 2.7 µM) of rTC-2, rTC-2Δ11, or E-64 used as control CP inhibitor. The time course of the fluorescence intensity for each assay was plotted using a GraphPad Prism 8.0.0 (GraphPad Software, Boston, Massachusetts USA, www.graphpad.com).

### 2.9 Cytotoxicity assays

To determine the inhibition of the trichomonal cysteine protease-dependent cytotoxicity on HeLa cells, *T. vaginalis* (CNCD 188) was grown for one week in trypticase-yeast extract-maltose (TYM) medium supplemented with 10% heat-inactivated adult bovine serum (HIBS) at 37°C, and for HeLa cell cultures, Dulbecco’s modified Eagle’s medium (DMEM) (Invitrogen-Gibco, Carlsbad, CA, USA) supplemented with 10% HIBS was used. HeLa cell monolayers were prepared by inoculating 3.5 x 10^4^ cells per well in flat bottom 96-well culture plates, incubated at 37°C for 24 h in a 5% CO_2_ atmosphere to allow the formation of confluent cell monolayers. The interaction of HeLa cells with *T. vaginalis* parasites was performed at a 1:5 ratio, as previously described (Alvarez-Sánchez et al., 2000). Briefly, parasites were previously incubated in interaction medium (DMEM: TYM, 2:1) without serum in the presence of rTC-2, rTC-2Δ11, E-64 as a positive inhibition control, or bovine serum albumin (BSA, an unrelated protein) as a negative inhibition control at 18 µM, 36 µM, and 54 µM concentrations. Then, the parasites were added to the HeLa cell monolayers and incubated for 2 h at 37°C and 5% CO_2_ atmosphere. Monolayer destruction was measured by a colorimetric method using crystal violet, as reported (Alvarez-Sánchez et al., 2000). Eluted stain from remaining cells was quantified with a VersaMax spectrophotometer (Molecular Devices) at a wavelength of 570 nm. Assays were conducted using technical and biological triplicates. Destruction of HeLa cell monolayer by untreated parasites was taken as 100% cytotoxicity and the inhibition percentages of each group were estimated, and an analysis of variance (Three-way ANOVA) was performed to evaluate significant differences between groups using the GraphPad Prism 8.0.0 (GraphPad Software, Boston, Massachusetts, USA, www.graphpad.com).

## 3 Results

### 3.1 *In silico* predictions of TC-2 and TC-2Δ11 interactions with target CPs

To determine whether the TC-2Δ11 protein, which lacks the first 11 amino acids of the N-terminus, could still interact with CPs and how these interactions compared with those of wild-type TC-2, *in silico* analyses were performed. These analyses were used to predict interactions between *T. vaginalis* CPs TvCP2 and TvCP39 and papain, a CP protein from an unrelated organism. Thus, the molecular dockings were done within the correct range of the predicted template modeling (pTM) score, the interface-predicted template modeling (ipTM) score, and the AlphaFold3 ranking score (**Supplementary Table S1**). The molecules were correctly validated for use in molecular docking in MolProbity (**Supplementary Table S2**). Moreover, in the Ramachandran plots (**Supplementary Figure S1**), the residues were within the favored and allowed regions for every molecule used in the molecular docking experiments except for the modeled TC-2 and TC-2Δ11 inhibitors. In the case of TC-2, two outlier residues were identified at G5 and V54, whereas in TC-2Δ11, one outlier was observed at V54 (**Supplementary Table S3**). Although these outliers indicate unfavorable amino acid positions, models were still validated. G5 is located in the unstructured N-Terminus (1-20 residues) of TC-2, and V54 is located at the end of the beta-sheet (S48-V54), in the Ramachandran’s plots, they were found too close to the allowed region limit and both amino acid residues have small side chains that may or may not restrict free rotation along the protein.

**Figure 1:**
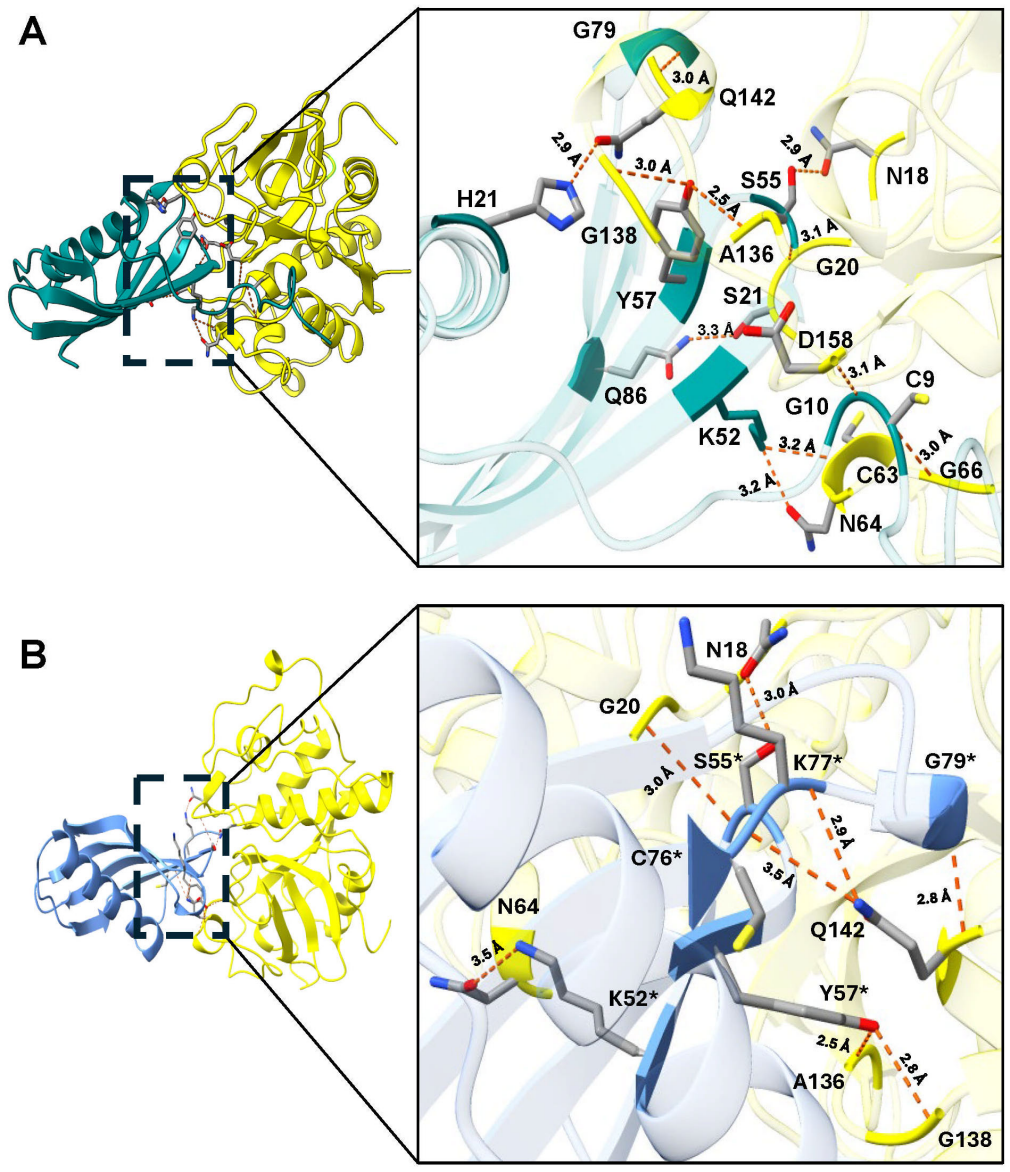
Molecular docking and interactions of the inhibitors TC-2 and TC-2Δ11 with papain. Three-dimensional modeling of the molecular docking of **(A)** TC-2 and papain and **(B)** TC-2Δ11 and papain. All the molecular docking interactions were performed using the AlphaFold3 server. The subsequent analysis, preparation, and visualization of the models were performed via ChimeraX software. The molecules are depicted using the following colors: TC-2 in teal, TC-2Δ11 in light blue, and papain in yellow. The enlargement shows the molecular distances of the interactions between proteins in angstroms. The TC-2Δ11 amino acid residues numbering refers to TC-2 sequence. The atoms that interact are shown in gray for carbon, blue for nitrogen, red for oxygen, white for hydrogen, and yellow for sulfur. *TC-2Δ11 sequence numbering corresponds to TC-2 sequence.

Molecular docking of rTC-2 with papain (**Figure 1A**) revealed 11 hydrogen bond interactions (**Table 1**). Two amino acids of rTC-2 (K52/S55) that interact with papain belong to the central domain of cystatin (QKVVSG) (Puente-Rivera et al., 2014). Binding affinity with ΔG~ (-13.1, -13.5) kcal/mol and *K_d_* ~ (1.2 × 10^-10^, 2.36 × 10^-10^) suggest that this binding can occur spontaneously (**Table 2**). In the case of the interaction between rTC-2Δ11 and papain (**Figure 1B**), three fewer hydrogen bonds interact between both proteins (**Table 1**), suggesting a lower affinity than rTC-2 and papain given that the binding affinity between rTC-2Δ11 and papain ΔG~ (-10.3, -7.4) kcal/mol and *K_d_* ~ (3.9 × 10^-6^ - 2.6 × 10^-8^) (**Table 2**).

**Table 1:** Interactions determined by the molecular dockings of the inhibitors TC-2 and TC-2Δ11 with papain^1^.

TC-2^2^	Papain	Distance (Å)	TC-2Δ11^2^	Papain	Distance (Å)
C9	G66	3.0			
G10	D158	3.1			
H21	Q142	2.9			
K52	C63	3.3			
K52	N64	3.2	K52	N64	3.5
S55	N18	2.9	S55	N18	3.0
S55	G20	3.1	S55	G20	3.0
Y57	A136	2.5	Y57	A136	2.5
Y57	G138	3.0	Y57	G138	2.8
			C76	Q142	3.5
			K77	Q142	2.9
G79	Q142	3.0	G79	Q142	2.8
Q86	S21	3.3			

^1^Hydrogen bond between amino acid residues determined by the molecular docking using the AlphaFold3 server ( **https://alphafoldserver.com/** ). ^2^The number of amino acid residue corresponds to the TC-2 sequence. Blank spaces no interaction detected.

**Table 2:** Metric analysis of molecular dockings of the inhibitors rTC-2 and rTC-2Δ11 with cysteine proteases.

Molecular docking	Prodigy^1^		Area Affinity^2^	
	ΔG (kcal/mol)	*K_d_*	ΔG (kcal/mol)	*K_d_*
TC-2 - Papain	-13.5	1.20E-10	-13.1	2.36E-10
TC-2 - TvCP2	-12.7	4.60E-10	-12.0	1.51E-09
TC-2 - TvCP39	-14.8	1.40E-11	-12.4	8.40E-10
TC-2Δ11 - Papain	-10.3	2.60E-08	-7.4	3.94E-06
TC 2Δ11 - TvCP2	-11.5	3.60E-09	-11.4	4.51E-09
TC-2Δ11 - TvCP39	-12.3	1.00E-09	-11.8	2.08E-09

^1^ https://rascar.science.uu.nl/prodigy/.^2^ https://affinity.cuhk.edu.cn/index.html. *K_d_*, disassociation constant.

**Figure 2:**
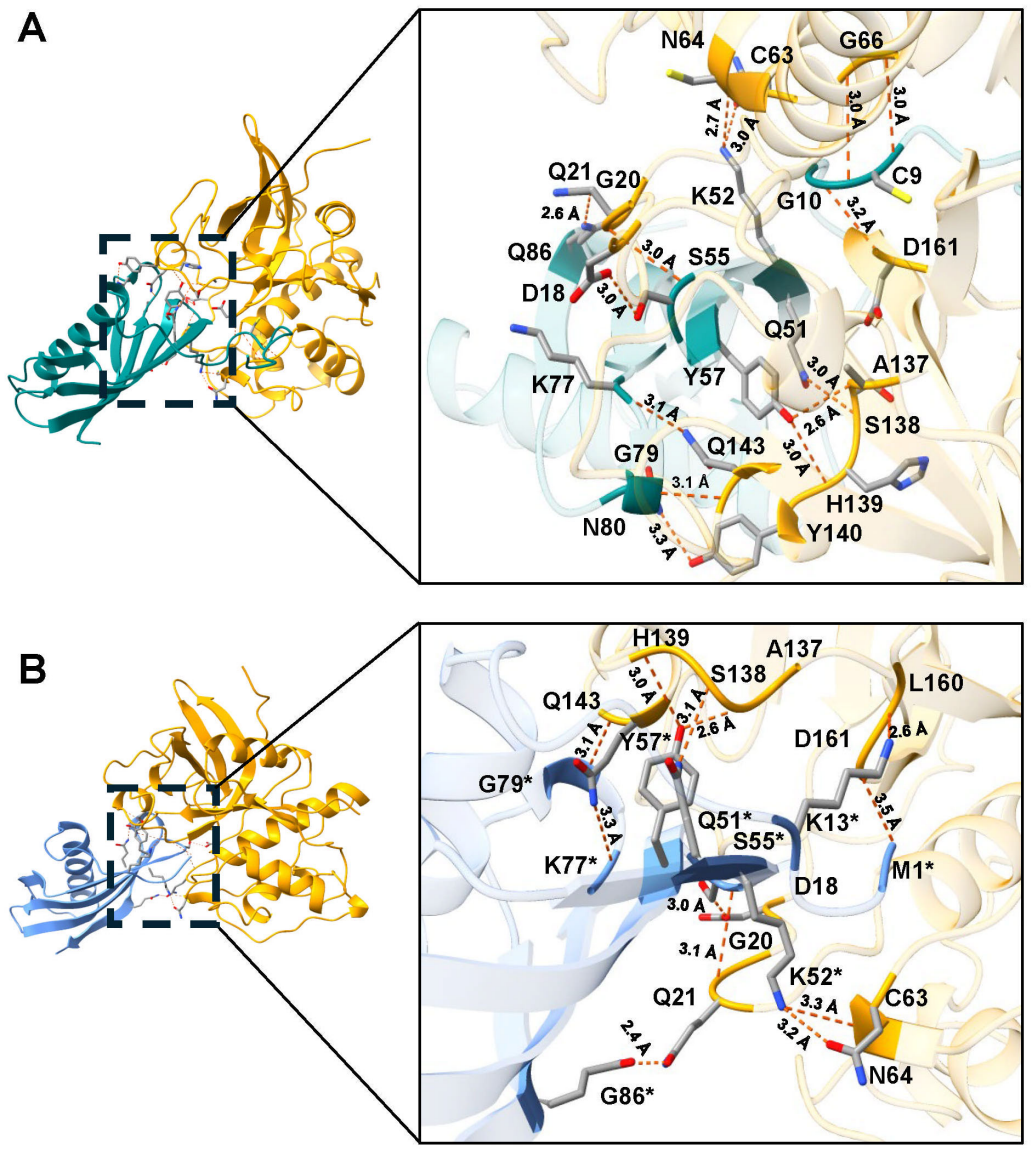
Molecular docking and interactions of the inhibitors TC-2 and TC-2Δ11 with TvCP2. Three-dimensional modeling of the molecular docking of **(A)** TC-2 and TvCP2 and **(B)** TC-2Δ11 and TvCP2. All the molecular docking interactions were performed via the AlphaFold3 server. The subsequent analysis, preparation, and visualization of the models were performed using ChimeraX software. The molecules are depicted using the following colors: TC-2 in teal, TC-2Δ11 in light blue, and TvCP2 in orange. The enlargement shows the molecular distances of the interactions between proteins in angstroms. The TC-2Δ11 amino acid residues numbering refers to TC-2 sequence. The atoms that interact are shown in gray for carbon, blue for nitrogen, red for oxygen, white for hydrogen, and yellow for sulfur. *TC-2Δ11 sequence numbering corresponds to TC-2 sequence.

**Figure 3:**
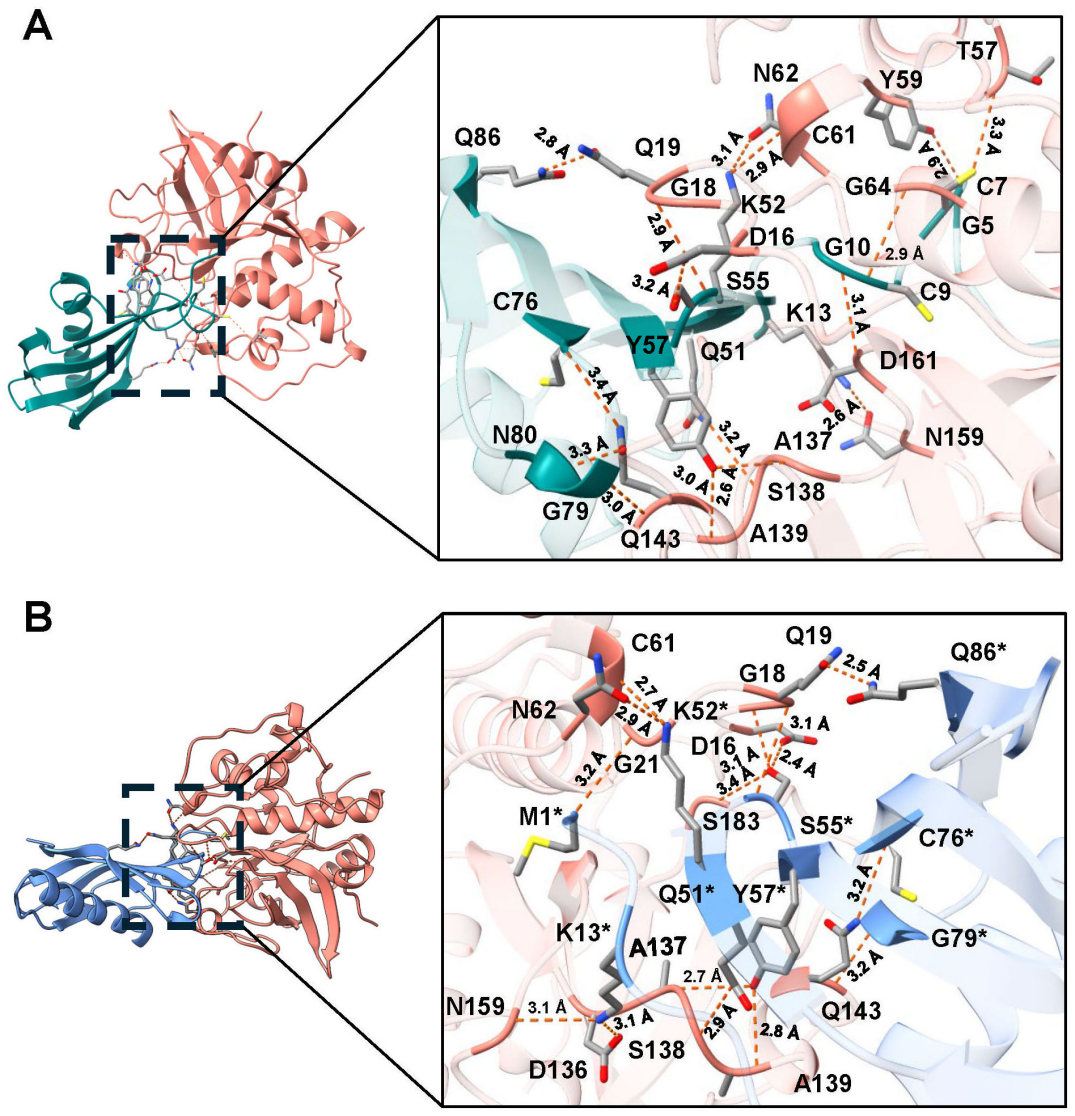
Molecular docking and interactions of the inhibitors TC-2 and TC-2Δ11 with TvCP39. Three-dimensional modeling of the molecular docking of **(A)** TC-2 and TvCP39 and **(B)** TC-2Δ11 and TvCP39. All the molecular docking interactions were performed via the AlphaFold3 server. The subsequent analysis, preparation, and visualization of the models were performed via ChimeraX software. The molecules are depicted using the following colors: TC-2 in teal, TC-2Δ11 in light blue, and TvCP39 in salmon. The enlargement shows the molecular distances of the interactions between proteins in angstroms. The TC-2Δ11 amino acid residues numbering refers to TC-2 sequence. The atoms that interact are shown in gray for carbon, blue for nitrogen, red for oxygen, white for hydrogen, and yellow for sulfur. *TC-2Δ11 sequence numbering corresponds to TC-2 sequence.

However, when rTC-2 interacted with *T. vaginalis* CPs, the number of interactions increased compared with those observed for rTC-2 binding with papain (**Tables 3**, **4**). Specifically, 14 interactions were noted between rTC-2 and TvCP2 (**Figure 2A**; **Table 3**), and 16 interactions were noted between rTC-2 and TvCP39 (**Figure 3A**; **Table 4**). The number of interactions of rTC-2Δ11 with *T. vaginalis* CPs was very close to that observed for rTC-2, with 12 interactions with TvCP2 (**Figure 2B**; **Table 3**), and 15 interactions found with TvCP39 (**Figure 3B**; **Table 4**). Thus, although there are fewer interactions between trichomonad CPs with rTC-2Δ11 than with rTC-2, recombinant inhibitors exhibit more interactions with *T. vaginalis* proteases than papain. Moreover, both rTC-2 and rTC2Δ11 also interacted with the Q51/K52/S55 conserved sites of *T. vaginalis* CPs (**Tables 3**, **4**).

**Table 3:** Interactions determined by the molecular dockings of the inhibitors TC-2 and TC-2Δ11 with *T. vaginalis* cysteine protease TvCP2^1^.

TC-2^2^	TvCP2	Distance (Å)	TC-2Δ11^2^	TvCP2	Distance (Å)
			M1	D161	3.5
			K13	L160	2.6
C9	G66	3.0			
C9	G66	3.0			
G10	D161	3.2			
Q51	S138	3.0	Q51	S138	3.1
K52	C63	3.0	K52	C63	3.3
K52	N64	2.7	K52	N64	3.2
S55	D18	3.0	S55	D18	3.0
S55	G20	3.0	S55	G20	3.1
Y57	A137	2.6	Y57	A137	2.6
Y57	H1391	3.0	Y57	H139	3.0
K77	Q143	3.1	K77	Q143	3.3
G79	Q143	3.1	G79	Q143	3.1
N80	Y140	3.3			
			Q86	Q21	2.4

^1^Hydrogen bond between amino acid residues determined by the molecular docking using the AlphaFold3 server ( **https://alphafoldserver.com/** ). ^2^The number of amino acid residue corresponds to the TC-2 sequence. Blank spaces no interaction detected.

**Table 4:** Interactions determined by the molecular dockings of the inhibitors TC-2 and TC-2Δ11 with *T. vaginalis* cysteine protease TvCP39^1^.

rTC-2^2^	TvCP39	Distance (Å)	rTC-2Δ11^2^	TvCP39	Distance (Å)
			M1	G21	3.2
K13	N159	2.6	K13	N159	3.1
			K13	D136	3.1
G5	Y59	2.9			
C7	T57	3.3			
C9	G64	2.9			
G10	D161	3.1			
Q51	S138	3.2	Q51	S138	2.9
K52	C61	2.9	K52	C61	2.7
K52	N62	3.1	K52	N62	2.9
S55	D161	3.2	S55	D16	2.4
S55	G18	2.9	S55	G18	3.1
		3.1	S55	G18	3.1
			S55	S183	3.4
Y57	A137	2.6	Y57	A137	2.7
Y57	A139	3.0	Y57	A139	2.8
C76	Q143	3.4	C76	Q143	3.2
			G79	Q143	3.2
N80	Y140	3.3			
Q86	Q19	2.8	Q86	Q19	2.5

^1^Hydrogen bond between amino acid residues determined by the molecular docking using the AlphaFold3 server ( **https://alphafoldserver.com/** ). ^2^The number of amino acid residue corresponds to the TC-2 sequence. Blank spaces no interaction detected.

In addition, the ΔG values for rTC-2 and rTC-2Δ11 were greater in terms of interactions with TvCP2 and TvCP39 than with papain, and the same trend was noted for *K_d_* (**Table 2**). This finding indicates a greater specificity of rTC-2 for *T. vaginalis* proteases. Moreover, the slightly lower affinity binding ΔG and *K_d_* values of rTC-2Δ11 with TvCP2 and TvCP39 indicate a lower affinity of the mutant toward the proteases; however, high values were also observed, indicating spontaneous binding (**Table 2**).

### 3.2 Recombinant TC-2 and TC-2Δ11 production at the bioreactor scale

**Figure 4:**
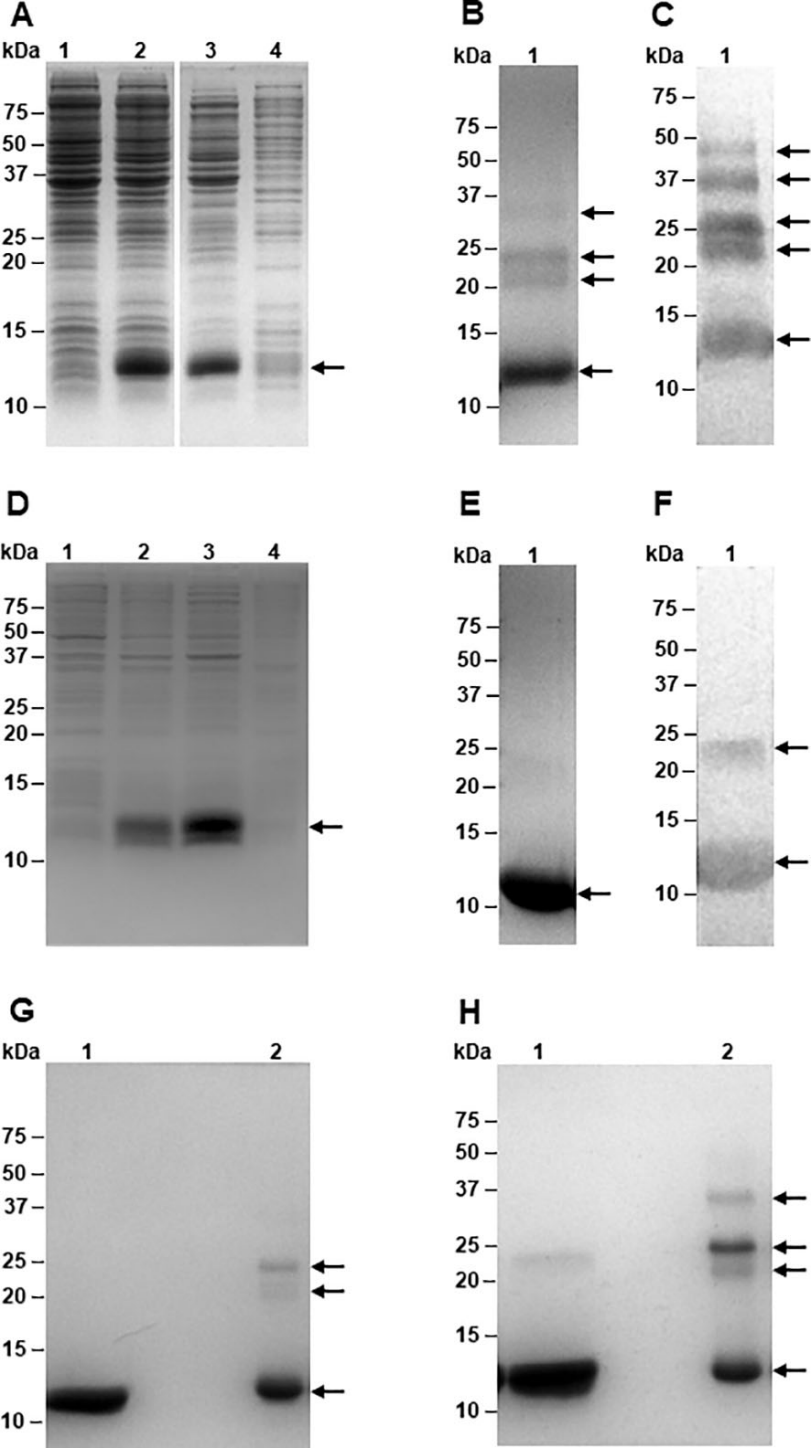
Expression and purification of recombinant TC-2 and TC-2Δ11 proteins. Electrophoretic profiles on SDS-PAGE of the expression of rTC-2 **(A)** and rTC-2Δ11 **(D)** before (lane 1) and after (lane 2) induction, soluble (lane 3), and insoluble (lane 4) fractions. SDS-PAGE **(B)** and Western-blot **(C)** of purified rTC-2. SDS-PAGE **(E)** and Western-blot **(F)** of purified rTC-2Δ11. SDS-PAGE under reducing **(G)** and nonreducing **(H)** conditions of purified rTC-2Δ11 (lane 1) and rTC-2 (lane 2), respectively. Arrows indicate the presence of the recombinant protein.

The recombinant expression of rTC-2 and rTC-2Δ11 was scaled up to a 2L bioreactor to do all assays with a single batch and determine their production yield at this scale. Recombinant TC-2 and TC-2Δ11 were expressed in *E. coli*, producing soluble protein (**Figures 4A, D**). A volumetric yield of ~990 mg of protein per liter of culture was obtained, and up to >95% purity was achieved after purification by IMAC (**Figures 4B, E**). Unlike rTC-2, rTC-2Δ11 did not aggregate (**Figures 4C–H**). Furthermore, even under nonreducing conditions, rTC-2Δ11 shows a faint band of aggregation (a dimer) compared with that of rTC-2 (**Figures 4G, H**). In addition, WB analysis with Rα-rTC-2 antibodies confirmed that each observed band belonged specifically to the rTC-2 protein and that there were no other bacterial-contaminating proteins (**Figures 4C, F**). The expression yield of ~ 1 g protein/L culture facilitated the purification process, it was sufficient to perform all the experiments using a single protein batch, and it is suitable for large scale production.

### 3.3 Biophysical properties of rTC-2 and rTC-2Δ11

#### 3.3.1 Molecular weight and protein size analysis using SEC and DLS assays

**Figure 5:**
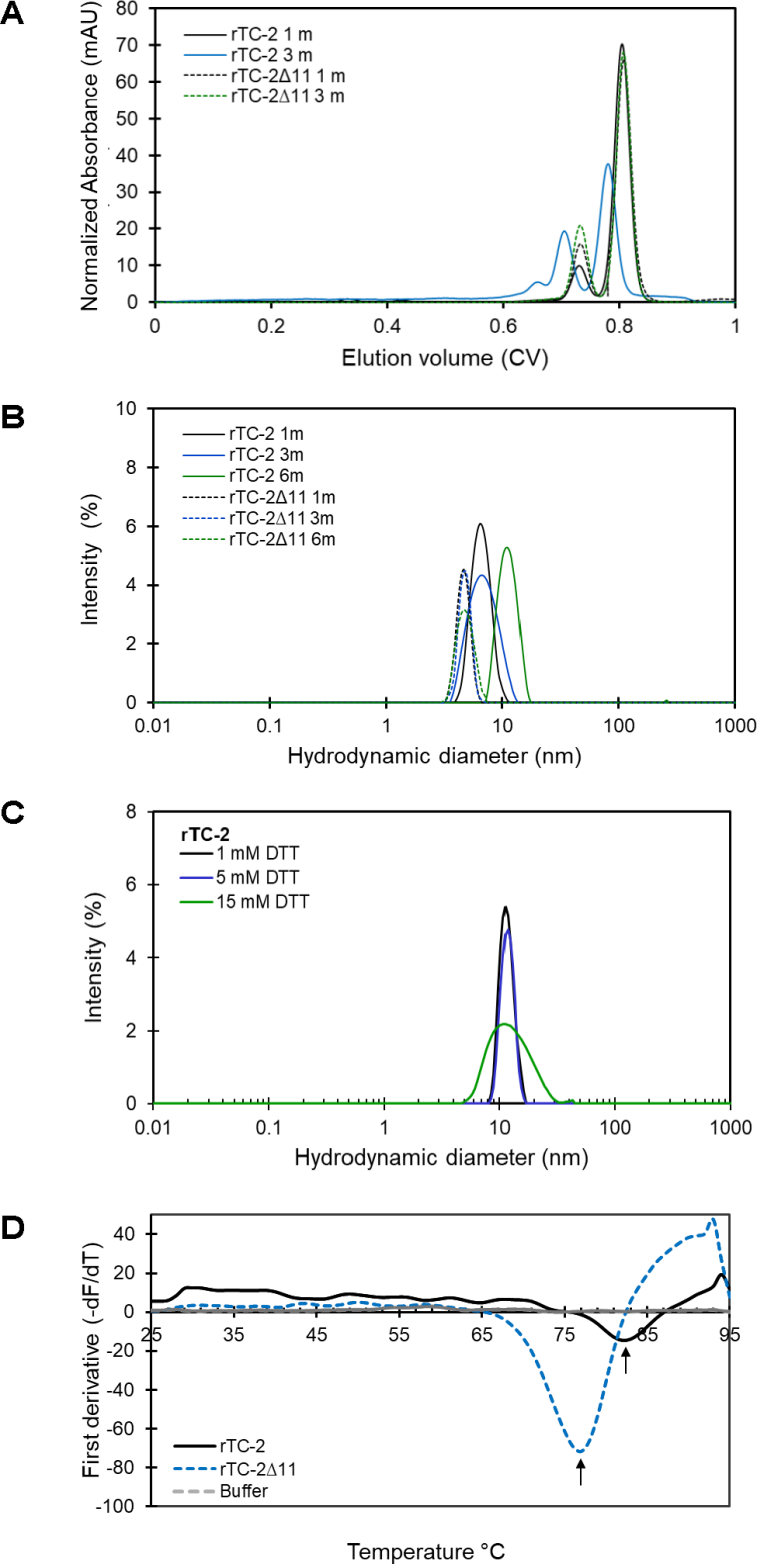
Biophysical characterization of rTC-2 and rTC2Δ11. **(A)** Analytical size exclusion chromatography (SEC) profiles of purified rTC-2 (continuous line) and rTC-2Δ11 (dotted line) after 1 to 3 months stored at 4°C. **(B)** Hydrodynamic diameter measurement for rTC-2 (continuous line) and rTC-2Δ11 (dotted line) determined using dynamic light scattering (DLS) of samples that were stored for 1, 3, or 6 months at 4°C. **(C)** Effect of DTT on the hydrodynamic diameter of samples stored at −20°C for a period longer than 1 year. Determinations were done at room temperature (23-25°C). Buffer was used as a negative control and Zetasizer data were corrected for buffer properties. The peaks represent more than 98% of the sample by mass. **(D)** Thermal shift (TS) assay of purified rTC-2 and rTC-2Δ11 at a final concentration of 20 µM of sample stored for 1 month at 4°C. The first derivative of the fluorescence−temperature curves for rTC-2 (continuous line) and rTC-2Δ11 (dashed line) is presented.

To evaluate and compare the aggregation-related characteristics of rTC-2 and rTC-2Δ11 proteins, their biophysical characterization was carried out by SEC and DLS assays to estimate their size and homogeneity. However, the high protein concentrations required by SEC analysis allowed us to analyze only samples from 1 and 3 months of storage. The results of the SEC analysis of both recombinant proteins were closer in the samples from 1 month of incubation since two peaks were observed (**Figure 5A**). The first peak corresponded to ~20-kDa, and the second peak corresponded to ~10-kDa, which corresponded to the dimer and the monomer, respectively, according to the electrophoretic profile (**Figure 4**). The dominant peak in both cases corresponded to the monomer (**Table 5**). The dimer peaks had molecular weights ranging from 19- to 28-kDa; however, for rTC-2 these molecular weight ranges increased after 3 months of storage, unlike rTC-2Δ11. This would indicate that the rTC-2 protein is more prone to form dimers depending on storage time compared to rTC-2Δ11 protein.

**Table 5:** Molecular weight estimation of rTC-2 and rTC-2Δ11 by SEC^1^.

	Storage time^2^ (Months)	Ve (CV)	Kav	Estimated MW (kDa)	Area (%)	Oligomers^3^
rTC-2 (13.6 kDa)^4^	1	0.81	0.698	9.7	83.2	M
		0.74	0.587	19.1	14.6	D
		0.43	0.095	ND	2.2	A
	3	0.78	0.651	13.0	62.2	M
		0.7	0.524	28.2	30.7	D
		0.65	0.444	45.7	7.1	T
rTC-2Δ11 (11.6 kDa)^4^	1	0.81	0.698	9.7	80.5	M
		0.73	0.571	21.1	19.5	D
	3	0.81	0.698	9.7	74.2	M
		0.73	0.571	21.1	25.8	D

^1^Size exclusion chromatography (SEC) was performed at room temperature (23-25°C) as described in the Materials and Methods section. Ve elution volume, CV column volume, MW molecular weight, Kav=(Ve-Vo)/(Vt-Vo) where Vo void volume and Vt total volume. ^2^Protein samples were stored at 4°C. ^3^Estimated molecular using a calibration curve (**Supplementary Figure S2**). Molecular weight corresponds approximately to Monomer (M), dimer (D), Tetramer (T), high molecular weight aggregates (A). ^4^Theoretical MW. ND not determined.

In the DLS measurements (**Table 6**), samples with longer storage times could be included. The results showed that samples of rTC-2 and rTC-2Δ11 with shorter storage times (1 month) revealed hydrodynamic diameters of 4.6 and 6.6 nm, respectively, with the largest diameter observed for rTC-2 (**Table 6**). The polydispersity values of 18% (rTC-2) and 13% (rTC-2Δ11) suggested that the mixture was homogeneous and comprised a single population. However, the hydrodynamic diameter gradually increased until it nearly doubled the initial Dh for rTC-2 after 3 months of storage, contrary to what happened with rTC-2Δ11, in which Dh increase was less pronounced and did not exceed 1 nm over the same time (**Figure 5B**). Since this increase in Dh is maintained for rTC-2 even after 6 months of storage, the tendency towards aggregation of rTC-2 through time was also corroborated by DLS, but not for rTC-2Δ11. For rTC-2 stored for more than 1 year, adding a reducing agent such as DTT did not significantly affect the reduction in Dh after incubation. In contrast, DTT’s presence caused a greater population size dispersion (**Figure 5C**). Together, these results demonstrate a greater propensity for aggregation of rTC-2, which cannot be improved even in the presence of reducing agents such as DTT, while this tendency is almost null for rTC-2Δ11 at times analyzed.

**Table 6:** Biophysical analysis of rTC-2 and rTC-2Δ11 by dynamic light scattering (DLS) and thermal shift (TS).

	Storage time^1^ (Months)	DTT (mM)	Dh^2^ (nm)	ZP^2^ (mV)	Tm^3^ (°C)	pI^4^	z@pH8.0^4^
rTC-2	1		6.6 ± 0.1	-14.1 ± 0.9	84 ± 1.3	7.11	-2.75
	3		7.4 ± 0.6	ND	ND		
	6		11.2 ± 0.3	ND	ND		
	> 12^5^	1	11.7 ± 0.5	ND	ND		
		5	11.8 ± 0.6	ND	ND		
		15	12.9 ± 0.5	ND	ND		
rTC-2Δ11	1		4.6 ± 0.04	-12.2 ± 0.4	77 ± 0.6	6.36	-3.25
	3		4.8 ± 0.1	ND	ND		
	6		4.9 ± 0.2	ND	ND		

^1^Protein samples were stored at 4°C and determinations were done at room temperature (23-25°C). ^2^Hydrodynamic diameter (Dh), mean ± SD of three data acquisition of two replicates in nanometers, and Zeta potential (ZP) in millivolts determined by DLS. ^3^Melting temperature (Tm) at 20 µM, mean ± SD of three replicates determined by TS assay. ND not determined.^4^Estimated isoelectric point (pI) and net charge at pH 8.0 (*z@pH8.0)* by using the CLC-Main Workbench 24.0.2 (Qiagen). ^5^rTC-2 sample was stored at -20°C for more than a year and thawed before DLS analysis.

#### 3.3.2 Protein stability analysis

To ascertain whether the absence of the 11 amino acid fragment of the NH_2_ terminus influenced the stability of rTC-2, the zeta potential and the thermal denaturation temperature (*Tm*) were determined for both rTC-2 and rTC-2Δ11 under identical conditions. **Table 5** shows the zeta potential values of -14.1 and -12.2 mV for rTC-2 and rTC-2Δ11, respectively. Despite the negative values and the observation that monomers predominated in the suspension by SDS−PAGE, these values were at the aggregation threshold for both proteins. Similarly, a TS assay was used to evaluate whether the absence of the initial 11 amino acids resulted in any alteration in the thermal denaturation temperature of the inhibitor, affecting protein stability. **Figure 5D** shows melting temperature values of 84°C and 77°C for rTC-2 and rTC-2Δ11, respectively. These data reflected an 7°C reduction in *Tm* when the amino-terminal region of 11 amino acids was removed from the original sequence, which does not compromise the stability of rTC-2Δ11 protein.

### 3.4 Protease activity inhibition by rTC-2 and rTC-2Δ11

**Figure 6:**
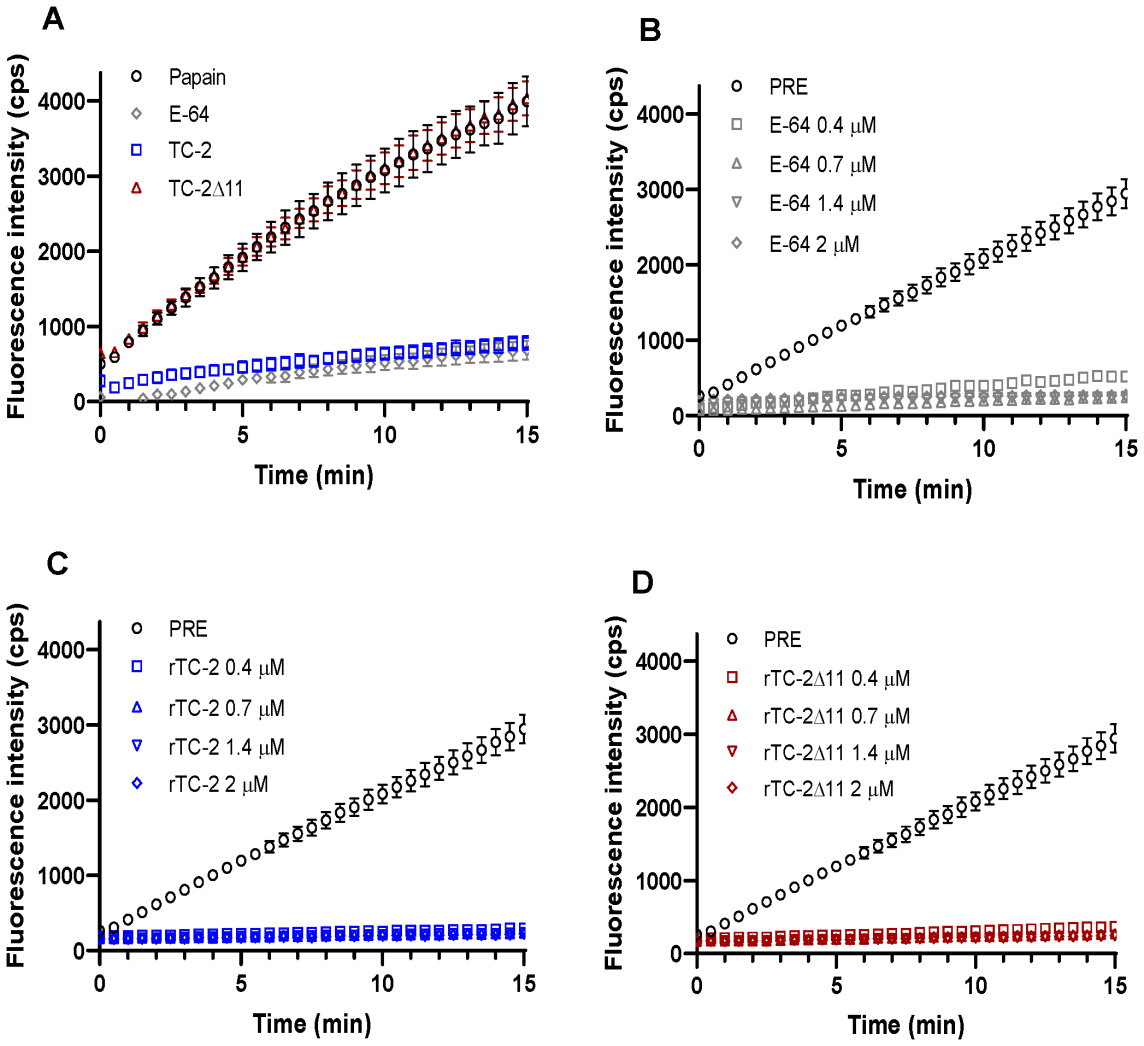
Inhibition of Papain and *T. vaginalis* PRE proteolytic activity by rTC-2 and rTC-2Δ11. The inhibition assays were performed using a fluorescent substrate as described in the Materials and Methods section. **(A)** Papain (2 ng/μL) (empty circle) activity in the presence or absence of rTC-2 (blue), rTC-2Δ11 (red), or E-64 (gray) at 0.7 μM. (**B–D)** Activity of protease-resistant extracts (PRE) (20 µg) from *T. vaginalis* (○) in the presence or absence of E-64 (gray), rTC-2 (blue), or rTC-2Δ11 (red) at 0.4 μM (□), 0.7 μM (Δ), 1.4 μM (∇), and 2 μM (◊).

To assess whether rTC-2Δ11 preserves its inhibitory capacity, inhibition assays were performed with papain as a model cysteine protease and with trichomonad PREs. Inhibition assays of papain proteolytic activity by rTC-2 and rTC-2Δ11 revealed that the inhibitory capacity of rTC-2Δ11 to papain activity was lost in the mutant as compared to rTC-2 in the range of concentrations tested. However, these differences were not observed when rTC-2 and rTC-2Δ11 were incubated with *T. vaginalis* PRE. Interestingly, even at the lowest concentrations tested, inhibition by the rTC-2Δ11 protein completely ablated the trichomonal proteolytic activity, similar to the inhibition caused by rTC-2 (**Figure 6**). These results suggest that the inhibition of CPs from other organisms, at least papain, is less specific than the inhibition of CPs from the same parasite. Furthermore, the essential sites for the interaction of the inhibitors with the trichomonad proteases were not located in the first amino acids of the N-terminal end. Thus, the four cysteines present at this end do not play an essential role in the inhibitory capacity toward *T. vaginalis* proteases.

### 3.5 Effects of rTC-2 and rTC-2Δ11 on *T. vaginalis* cytotoxicity

**Figure 7:**
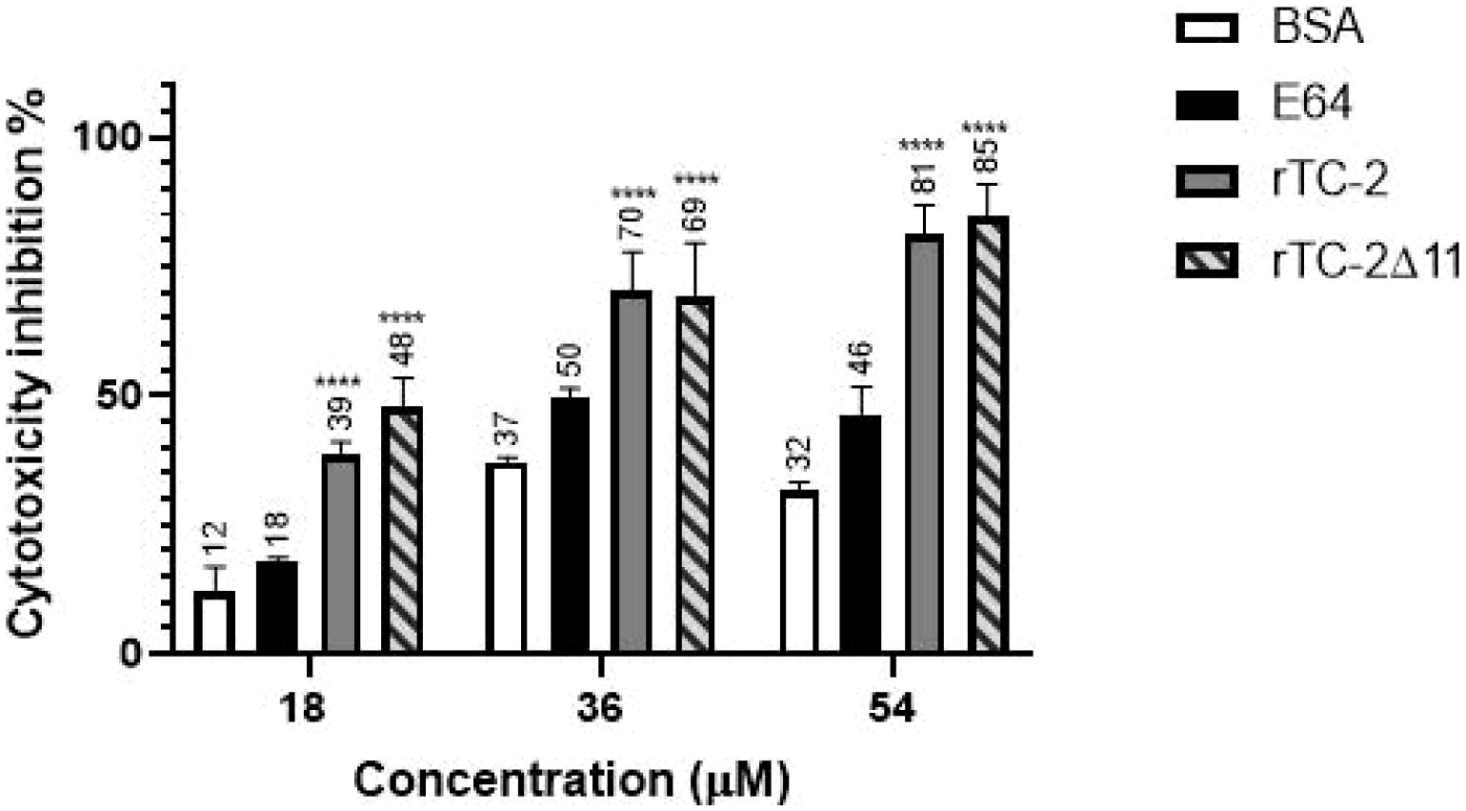
Inhibition of *T. vaginalis* cysteine protease-dependent HeLa cell cytotoxicity by rTC-2 and rTC-2Δ11. Inhibition of the cytotoxic effects of *T. vaginalis* on HeLa cell monolayers incubated with increasing concentrations (18 µM, 36 µM, and 54 µM) of rTC-2, rTC-2Δ11, BSA (unrelated protein) and E-64 (a CP specific inhibitor). ****Significant statistical difference or P value= <.0001. See the Materials and Methods section for details.

Cytotoxicity analyses were performed to verify whether rTC-2Δ11 also confers protection to HeLa cell monolayers from *T. vaginalis* protease activity. This is the case rTC-2, as its protective activity has already been reported (Puente-Rivera et al., 2014). **Figure 7** shows that both recombinant inhibitors significantly decreased the cytotoxicity levels at all concentrations tested and at all times compared with those of BSA, an unrelated protein, which was used as a control (**Figure 7**). Notably, both rTC-2 and rTC-2Δ11 had similar protective effects on HeLa cell monolayers against *T. vaginalis* protease activity. These proteins provided up to 81% and 85% protection in a concentration-dependent manner. These results indicate that, under the conditions tested, rTC-2Δ11 retains its CP inhibitory ability and protective role over HeLa cell monolayers without the first 11 amino acids. Thus, the N-terminus of TC-2 does not contain the amino acids essential for the inhibitory capacity of TC-2 against *T. vaginalis* CPs.

## 4 Discussion

In this study, TC-2 inhibitor was further characterized at the bioinformatic, biophysical, biochemical, and functional levels by analyzing the effects of four cysteines located at the N-terminus on the aggregation and inhibition of parasite CPs and protection of the HeLa cell monolayer destruction by *T. vaginalis*.

First, molecular docking analyses were performed to analyze whether a protein that lacks 11 amino acids at the N-terminus, where four of the five cysteines are located, affects its ability to inhibit the proteolytic activity of cysteine proteases, such as papain as well as TvCP39 and TvCP2 proteases of *T. vaginalis*. Thus, the *in silico* molecular docking analyses of rTC-2Δ11 with papain revealed fewer interactions than that noted for rTC-2. However, many hydrogen bonds are still formed with the papain and *T. vaginalis* CPs. Both the rTC-2 and rTC2Δ11 inhibitors presented exposed loops, which potentially allow better binding with CPs since the mechanism by which protein inhibitors inhibit papain-like proteases occurs when one binding site of the inhibitor is partially exposed to the solvent of the proteases (Tušar et al., 2021). Additionally, six of the interacting amino acids (QKVVSG) of TC-2 belong to the cystatin central domain, which interacts with the three proteases studied (papain, TvCP2, TvCP39). According to previous reports, this sequence is essential for CP inhibitory function and is conserved among the cystatin superfamily proteins (Puente-Rivera et al., 2014). Notably, this essential sequence is located in the N-terminus, which, together with a conserved glycine residue and a C-terminal PW hairpin loop, forms the CP interaction site. These three elements direct the cystatin molecule into the active site cleft of the CP (Sajid and McKerrow, 2002). This finding explains why removing 11 amino acids from the N-terminus resulted in fewer interactions between rTC-2Δ11 and the analyzed proteases.

Regarding the amino acids of the proteases that interact with TC-2, G66 was identified as an interacting residue in TvCP2. In contrast, Q19 interacts with TC-2 and TC-2Δ11 in TvCP39; this could be a conserved sequence since Q19 together with D158 was also found to be present in the selective cathepsin L inhibitor CLIK-148. This inhibitor interacts with papain residues Q19, G66, and D158 via hydrogen bonding; C25 via covalent bonding; and W177 and S205 via hydrophobic interactions (Beavers et al., 2008). Similarly, the number of interactions between TC-2 and TC-2Δ11 was greater than the number of interactions between TC-2 and papain, suggesting the specificity of this inhibitor. This specificity is potentially observed because the polypeptide chains of family 2 cystatins contain three segments directly involved in inhibiting CPs. The wedge-shaped binding region contains two loop segments that have been conserved during the evolution of family 2 cystatins, as is the case for Q55-G59 and P105-W106 from human cystatin C (Margis et al., 1998), which are the amino acids S55, F59, and P78 in TC-2.

These results showed that the N-terminal region of 11 amino acids, which lacks a sequence structure, may not be necessary for the inhibitory function of TC-2. Instead, it may be involved in rapid multimerization or aggregate formation. Although the rTC-2 inhibitor could be expressed in a soluble form with a high yield, downstream manipulations, and storage present challenges because of the propensity of the protein to form dimers or multimers upon processing and storage. Thus, when the rTC-2Δ11 protein was produced, soluble expression and protein yield were unaffected nor decreased. Furthermore, the removal of an 11-amino acid fragment containing four cysteines prevented immediate dimerization in rTC-2Δ11, in contrast to rTC-2. However, it cannot be excluded that, *in vivo*, the four N-terminal cysteines could play a role in the CP-TC2 interaction and proteolytic activity regulation.

With respect to biophysical characterization, the SEC elution profiles of rTC-2 and rTC-2Δ11 showed two prominent peaks representing the monomer and dimer. The formation of dimers could also be associated with the fifth cysteine of TC-2, which, according to the 3D model, is exposed to the solvent. Additionally, the formation of a structure called the swapping domain, which refers to the reversible exchange of domains between two monomers of the same cystatin and serves as a mechanism of regulation of its inhibitory function, has been reported for cystatin-type proteins (Sanders et al., 2004; Simpson et al., 2024). Dimer formation would be expected as a result of this phenomenon, as observed in rTC-2Δ11 throughout the duration of storage, given that the conserved region responsible for inhibiting protease activity and presumably involved in dimerization was not modified (Mascarenhas and Gosavi, 2017). This dimerization phenomenon would also result in a nonglobular molecule, explaining why, according to the DLS measurements, the diameter of both recombinant TC-2 proteins is much larger than expected. Although the DLS analyses cannot discriminate between the effect of shape and the effect of oligomerization, they do allow us to demonstrate the trend of the increase in the hydrodynamic diameter of the molecule over time. Additionally, the dispersion values (Pd), which represent the width of the particle size distribution, showed that the dispersion for rTC-2 is more significant than that for rTC-2Δ11, indicating oligomerization or aggregation in rTC-2. This oligomer formation of rTC-2 was also observed via SEC analysis, confirming the presence of high-molecular-weight aggregates that remain even under reducing conditions, unlike the rTC-2Δ11 behavior.

The TS assay was used to assess another stability parameter associated with thermal denaturation. The Tm of both proteins was greater than 75°C, indicating a high tolerance to thermal denaturation, despite a decrease of 7°C in the Tm for rTC-2Δ11, with a Tm comparable to that of cystatins from other organisms with reported Tm values ranging from 66°C for human stefin B to 115°C for chicken cystatins (Zerovnik et al., 1997; Yadav et al., 2013; Júnior et al., 2017; Zalar et al., 2019). These results showed that removing the 11 residues in the N-terminus decreased the propensity for multimerization without compromising the stability of TC-2Δ11 or the inhibitory capacity toward trichomonad CPs.

Concerning the role of the absence of the N-terminus in the inhibition of parasite proteases, it was interesting to observe contrasting results between the inhibition of papain and *T. vaginalis* PRE since the rTC-2Δ11 protein completely lost its inhibitory capacity with papain. This would result from the fewer interactions (up to 50% less) observed in the *in silico* analysis between rTC-2 and papain than between rTC-2 and TvCP2 or TvCP39. Differences in affinities between rTC-2Δ11 and rTC-2 toward papain have also been observed for human cystatin A lacking the six N-terminal amino acids, which exhibits a lower affinity for papain (Pol et al., 1995). The role of the N-terminus of cystatins in the interaction with papain has already been described in the literature. For example, the crystal structure of chicken cystatin with papain revealed that the N-terminal trunk of cystatin is involved in blocking the reactive site of the target protease. Along with a loop, this region stabilizes the inhibitor structure through interactions with a β-sheet and an α-helix, which helps maintain the integrity of the binding complex (Tušar et al., 2021).

In contrast to papain, the inhibitory capacity of rTC-2 against *T. vaginalis* PRE was not affected by the absence of the first 11 amino acids in the N-terminus of rTC-2Δ11. Differences in the inhibitory effects of parasite cystatins on CPs from other organisms have been reported even for complete cystatins. For example, *Giardia intestinalis* cystatins strongly inhibit parasite proteases but exhibit a lower degree of inhibition of human cathepsin B (Liu et al., 2019); this could be because cystatins exhibit significant specificity and diversity among different species, reflecting their evolutionary adaptations and functional roles. In plants, animals, and parasites, cystatins have evolved unique sequences, structures, and expression patterns that enable them to perform specialized functions, particularly in regulating protease activity and modulating immune responses. This diversity underscores the importance of cystatins in various biological processes and their potential as targets for therapeutic interventions (Margis et al., 1998; Cuesta-Astroz et al., 2014).

Finally, it was also confirmed that rTC-2Δ11 retained its ability to reduce the cytotoxic effect of *T. vaginalis* proteases on HeLa cells under the conditions tested. In addition, a concentration-dependent protective effect was observed for both TC-2 recombinant proteins, which agrees with that previously reported for rTC-2 (Puente-Rivera et al., 2014). This effect may be because cystatins inhibit parasite CPs, which are vital for various stages of parasite life cycles, including development, host tissue invasion, and migration. Therefore, inhibiting these enzymes can disrupt these processes, potentially leading to reduced parasite survival and reproduction. Additionally, inhibiting these proteases can hinder the ability of the parasite to obtain necessary nutrients, which can affect its growth and virulence (Rascon and McKerrow, 2013). For example, the inhibition of proteases from *Streptococcus pneumoniae* can block pathogen immune response evasion and tissue invasion, reducing infection and virulence, which are protease-related functions (Wang et al., 2020). However, as has been reported, TC-2 inhibits the papain-like CPs such as TvCP39 but not the legumain-like CPs (Puente-Rivera et al., 2014). Therefore, the inhibition of the proteolytic activity of PRE observed and reported in this work by rTC-2 and rTC-2Δ11 are limited to the papain-like CPs and cytotoxicity of *T. vaginalis* on HeLa cells depending on it. Whether lack of the first 11 amino acid residues in rTC-2Δ11 wider its inhibition spectrum to legumains is something that should be tested, as well as their effect on the other virulence properties of *T. vaginalis*, such as adhesion and hemolysis, among others.

## 5 Conclusions

The results of the *in silico* analysis and the inhibition assays were consistent, as the interaction capacity of rTC-2 and rTC-2Δ11 with papain and *T. vaginalis* proteases and the number of interactions found in each case were comparable. It was also found that, at least *in vitro*, the first 11 amino acids of the N-terminus of TC-2 were not required to inhibit *T. vaginalis* CPs or for CP-dependent HeLa cell cytotoxicity. In summary, deleting the four cysteines of the N-terminus of TC-2 improved recombinant production by preventing aggregation without abrogating its ability to inhibit *T. vaginalis* CPs, suggesting that rTC-2Δ11 has potential therapeutic applications against trichomoniasis.

## Data Availability

The original contributions presented in the study are included in the article/**Supplementary Material**. Further inquiries can be directed to the corresponding author.
