# Supplementary material for: Trichocystatin-2 from Trichomonas vaginalis: role of N-terminal cysteines in aggregation, protease inhibition, and trichomonal cysteine protease-dependent cytotoxicity on HeLa cells
Source: Front Parasitol. 2025 Mar 18;4:1512012. doi: 10.3389/fpara.2025.1512012 (PMC11959277; doi:10.3389/fpara.2025.1512012)
Supplement: Supplementary file 1 [file DataSheet1.pdf]

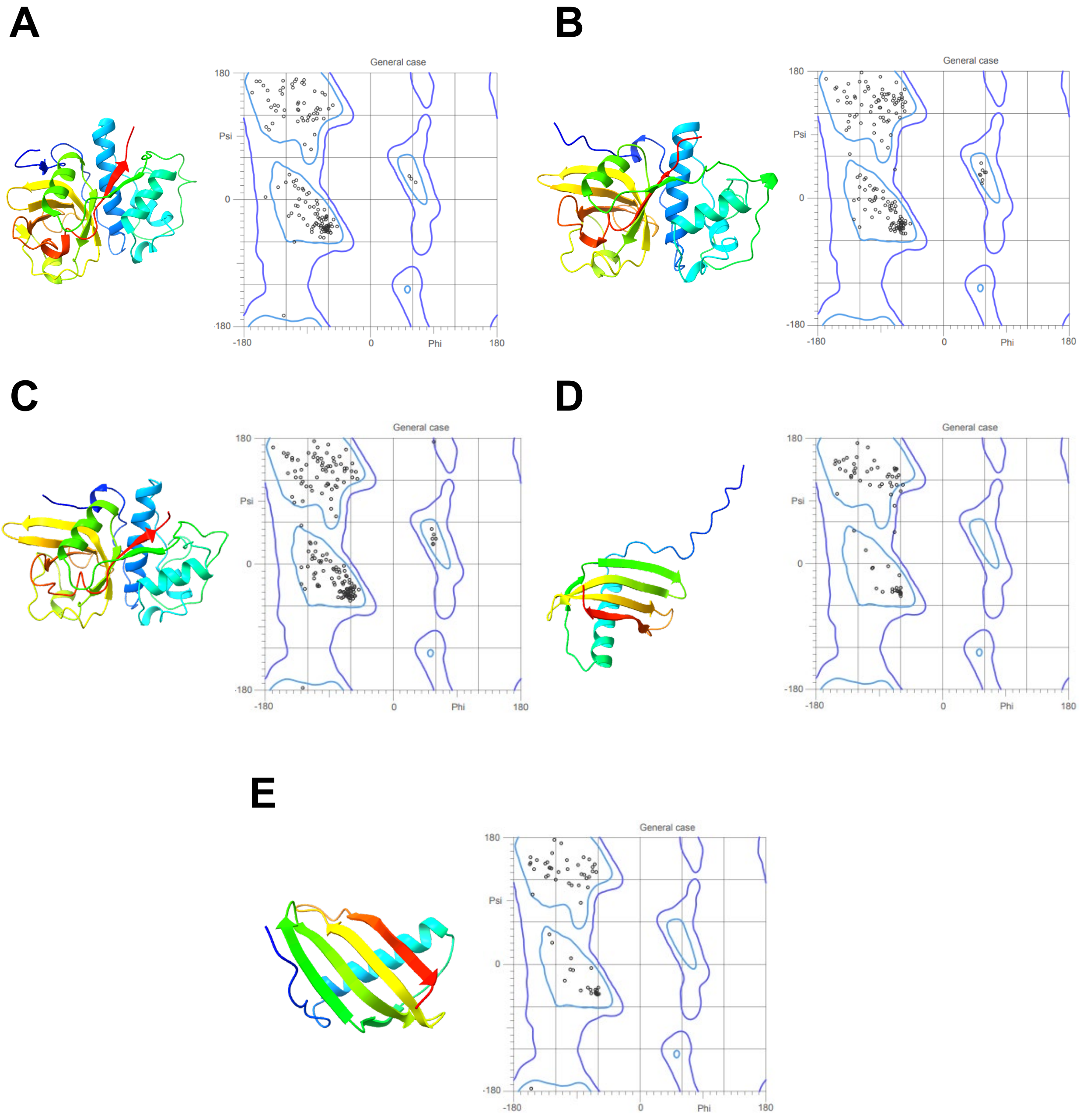


**Figure S1. 3D structure and Ramachandran plots of the molecules used in molecular docking by AlphaFold3.** A) papain, B) TvCP2, C) TvCP39, D) TC-2 and E) TC-2Δ11. The light blue regions indicate the most favored region, the dark blue regions indicate allowed regions, and the white regions indicate disallowed regions. All Ramachandran plots were made by Molprobity.

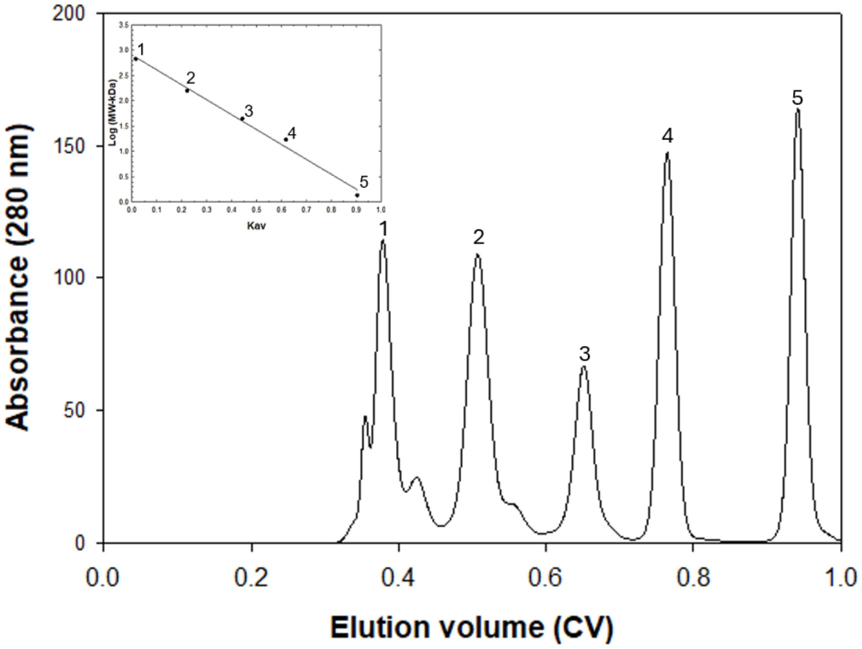


**Figure S2**. **Calibration of SEC column.** Chromatographic profile of the elution of gel filtration standards (Bio-Rad #1511901): thyroglobulin (1), bovine γ-globulin (2), chicken ovalbumin (3), equine myoglobin (4), and vit B12 (5) in a Superdex 200 pg HiLoad 26/600 column. Inset shows the linear regression of the Kav values vs. Log (MW).
