# Supplementary material for: Trichocystatin-2 from Trichomonas vaginalis: role of N-terminal cysteines in aggregation, protease inhibition, and trichomonal cysteine protease-dependent cytotoxicity on HeLa cells
Source: Front Parasitol. 2025 Mar 18;4:1512012. doi: 10.3389/fpara.2025.1512012 (PMC11959277; doi:10.3389/fpara.2025.1512012)
Supplement: Supplementary file 2 [file DataSheet2.pdf]

Supplementary Tables

Table S1. Confidence metrics and accuracy of modeled structures by AlphaFold3[1].

Molecular Docking	Ranking score	Predicted template modeling (pTM)	Interface predicted template modeling: (ipTM)
**TC-2-Papain**	0.87	0.90	0.81
**TC-2-TvCP2**	0.92	0.93	0.90
**TC-2-TvCP39**	0.92	0.93	0.89
**TC-2Δ11-Papain**	0.85	0.91	0.84
**TC-2Δ11-TvCP2**	0.90	0.93	0.89
**TC-2Δ11-TvCP39**	0.89	0.93	0.88

[1]https://alphafoldserver.com/

Table S2. Confidence metrics of structures determined by MolProbity[1].

Confidence metrics	TC-2	TC-2Δ11	TvCP2	TvCP39	Papain
**Clashscore (all atoms)**	4.38	3.11	7.11	6.97	4.31
**Poor rotamers (%)**	0	0	0	0	2.94
**Favored rotamers (%)**	100	100	98.84	97.70	95.29
**Ramachandran outliers (%)**	2.25	1.25	0	0	0
**Ramachandran favored (%)**	93.26	98.75	98.13	98.13	98.10
**Rama distribution Z-score**	-0.60 ± 0.81	0.93 ± 0.94	-1.29 ± 0.50	-1.09 ± 0.53	-1.07 ± 0.50
**MolProbity score**	1.65	1.10	1.39	1.38	1.57
**Cβ deviations (%)**	0	0	0	0	0
**Bad bonds (%)**	0	0	0	0	0
**Bad angles (%)**	0	0	0.22	0.27	0

[1]http://molprobity.biochem.duke.edu/

Table S3. Metrics of Ramachandran plots determined by MolProbity.

	Residues		
**Protein**	**favored regions (%)**	**allowed regions (%)**	**Outliers (phi, psi)**
**Papain**	98.1	100.0	No
**TvCP2**	98.1	100.0	No
**TvCP39**	98.1	100.0	No
**TC-2**	93.3	97.8	G5 (-63.5, 90.6), V45 (-129.9, -136.7)
**TC-2Δ11[2]**	98.8	98.8	V45 (-133.3, -147.1)

[1]http://molprobity.biochem.duke.edu/ .[2] The numbers on amino acid residues of TC-2Δ11 are referred to the TC-2 sequence.
